# Ecosystem Service Values Changes in Response to Land-Use/Land-Cover Dynamics in Dry Afromontane Forest in Northern Ethiopia

**DOI:** 10.3390/ijerph16234653

**Published:** 2019-11-22

**Authors:** Negasi Solomon, Alcade C. Segnon, Emiru Birhane

**Affiliations:** 1 Department of Land Resources Management and Environmental Protection, Mekelle University, P.O. Box 231, 7000 Mekelle, Ethiopia; solomonnegasi@gmail.com (N.S.); emiru.birhane@mu.edu.et (E.B.); 2 Faculty of Agronomic Sciences, University of Abomey-Calavi, 01 BP 526 Cotonou, Republic of Benin

**Keywords:** forest ecosystems, ecosystem service values, land-use/land-cover change, Afromontane forest, ecosystem services, Ethiopia

## Abstract

Despite their importance as sources of ecosystem services supporting the livelihoods of millions of people, forest ecosystems have been changing into other land use systems over the past decades across the world. While forest cover change dynamics have been widely documented in various ecological systems, how these changes affect ecosystem service values has received limited attention. In this study we assessed the impact of land-use/land-cover dynamics on ecosystem service values in dry Afromontane forest in Northern Ethiopia. We estimated ecosystem service values and their changes based on the benefit transfer method using land cover data of the years 1985, 2000, and 2016 with their corresponding locally valid value coefficients and from the Ecosystem service valuation database. The total ecosystem service values of the whole study area were about USD 16.6, 19.0, and 18.1 million in 1985, 2000, and 2016, respectively. The analyses indicated an increase in ecosystem service values from 1985 to 2000 and a decrease in ecosystem service values from 2000 to 2016. Similarly, the contribution of specific ecosystem services increased in the first study period and decreased in the second study period. The findings highlight how forest cover dynamics can be translated into changes in ecosystem service values in dry Afromontane forest ecosystems in Northern Ethiopia and showed how specific ecosystem services contributed to the observed trends. The findings also illustrated the temporal heterogeneity in the impacts of land-use/land-cover dynamics on values of ecosystem services. The findings can serve as crucial inputs for policy and strategy formulations for the sustainable use and management of forest resources and can also guide the allocation of limited resources among competing demands to safeguard the ecosystems that offer the best-valued services.

## 1. Introduction

Forest ecosystems are important sources of ecosystem services (ES) and goods for millions of people [1]. Despite their importance, forest ecosystems and covers have been changing across the world over the past decades due to both direct and indirect drivers, such as agricultural expansion (both commercial and subsistence agriculture), demographic pressure, timber extraction and logging, fuelwood collection and charcoal production, uncontrolled fires, livestock grazing, mining, urbanization, and infrastructural development [2,3,4,5,6,7,8,9]. Forest ecosystem changes and degradation and their drivers have been widely documented in various ecological settings [3,4,7,8,9,10,11], including in Ethiopia [12,13,14].

While forest ecosystems changes have been extensively investigated in various ecological contexts across the globe, our understanding of the implications of these changes on ES values and subsequently the livelihoods of people who depend on those ES are scanty, particularly in Afromontane ecosystems. Over the past two decades, there has been a growing body of literature on the impact of land-use/land-cover change on ecosystem services values from global [15,16,17] to landscape scales [18,19,20,21,22,23]. In Ethiopia, several studies have been carried out to estimate forest cover change [24,25,26,27,28,29]. However, studies on the effects of land-use/land-cover changes on ecosystem services are scant, except very few studies in the central highlands [19,20] and the Blue Nile basin of Ethiopia [30], overlooking other important ecosystems such as Afromontane ecosystems. For instance, Kindu et al. [19] developed modified value coefficients for 11 biomes for the central highlands of Ethiopia using expert knowledge of the study landscape conditions and other studies, mainly from The Economics of Ecosystems and Biodiversity (TEEB) valuation database [31,32]. 

Afromontane vegetation covers more than 50% of the highlands in Ethiopia with a high diversity of plant species and is one of the prominent biodiversity havens in the country [33]. Afromontane forests provide a range of ecosystem services, including the provision of diverse habitats for fauna and fodder for livestock, watershed protection (e.g., groundwater regulation, flood control, and soil erosion prevention and control), non-timber forest products, and climate change mitigation [34,35,36]. However, a thorough understanding of changes in ecosystem service values due to forest cover change through quantitative knowledge is limited in Afromontane ecosystems. 

Assessing changes in ecosystem service values in response to land-use/land-cover change is crucial to generate awareness and policy and decision making on the allocation of limited resources among competing demands to safeguard the ecosystems that offer the best-valued services. This study contributes to the growing body of knowledge on the impacts of land-use/land-cover changes on ecosystem service values, with insights for the dry Afromontane forest ecosystems in Northern Ethiopia. 

**Figure 1:**
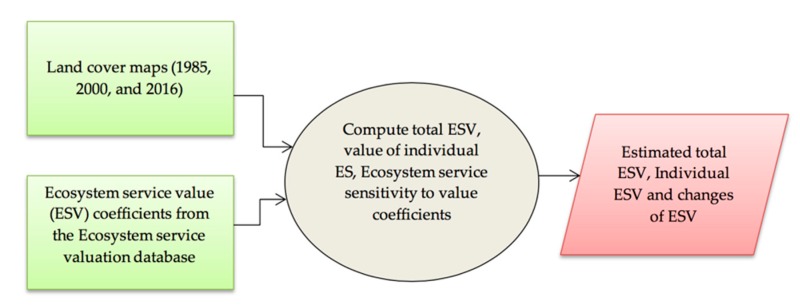
Analytical framework describing the methodological approach of the study. ES: ecosystem services.

In this study we investigated how forest land cover change dynamics affect the ES values in Wujig Mahgo Waren forest in Northern Ethiopia. Specifically, we estimated ecosystem service values of land cover classes over the past three decades (1985–2016) and linked land use dynamics to changes in ES values. Based on land-use/land-cover data of 1985, 2000, and 2016 (see Solomon et al. [37]), we estimated ecosystem service values and their changes based on the benefit transfer method. The corresponding locally valid ecosystem service value coefficients for each year and land use class were retrieved from the Ecosystem service valuation database. The analytical framework of the study is depicted in Figure 1.

## 2. Materials and Methods

### 2.1. Study Area

**Figure 2:**
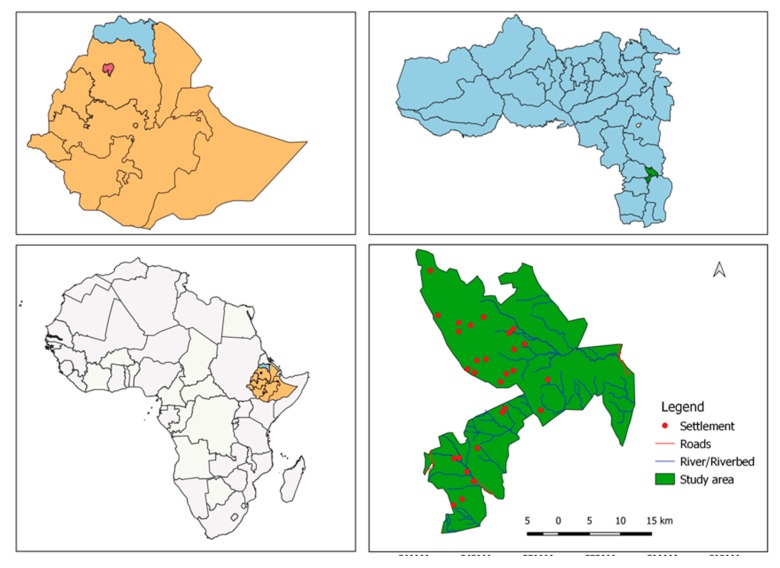
Map of Wujig Mahgo Waren forest in Tigray region, Northern Ethiopia.

Covering a total area of 17,000 ha, the Wujig Mahgo Waren forest (12°47′–13°02′ N to 39°26′–39°39′ E) is a natural forest in the southern Tigray region in Northern Ethiopia (Figure 2). It is a state forest found within four districts, namely, Alaje, Enda-Mehoni, Hinatalo-Wajerat, and Raya-Azebo. The area is characterized by an undulating landscape where dense forest is found on the hillsides and the valleys are left as cultivated areas and scattered bushlands [37]. The elevation ranges from 1404 to 3924 m above sea level. The climate is a semiarid type, with mean annual temperature ranging between 8 and 25 °C, and mean annual rainfall of 800 mm distributed between June and September (main rainy season) and February to May (short rainy season). The types of soils are Vertisols, Cambisols, Fluvisols, Regosols, and Leptosols [38].

The forest is composed of indigenous and exotic species, mainly *Acacia abyssinica* [Hochst. ex] Benth, *Eucalyptus globulus* Labill, *Eucalyptus camaldulensis* Dehnh, Juniperus procera Hochst. ex Endl, *Olea europaea* ssp. *africana* Mill, *Podocarpus falcatus* (Thunb.) Mirb, *Dodonea angustifolia* L.F., *Combretum molle* R.Br. ex G. Don, *Cadia purpurea* (G. Piccioli) Aiton and *Opuntia ficus-indica* (L.) Mill. The Wujig Mahgo Waren forest is one of the remnants of the dry Afromontane forest in Northern Ethiopia which continues to provide essential services for the livelihoods of the people. It provides a range of ecosystem services including the provision of diverse habitats for fauna and fodder for livestock; non-timber forest products; watershed protection including groundwater regulation, flood control, and soil erosion prevention and control; and carbon sequestration. It is also the main source of raw material for housing and agricultural implements. The community in the study area practice rain-fed subsistence-mixed farming. Small-scale farmers with an average landholding size of less than one hectare per household dominate the area. Southern Tigray is known for its high potential for wheat, barley, faba bean, and maize production as well as its rich livestock.

### 2.2. Data Used

#### 2.2.1. Land Cover Data

**Figure 3:**
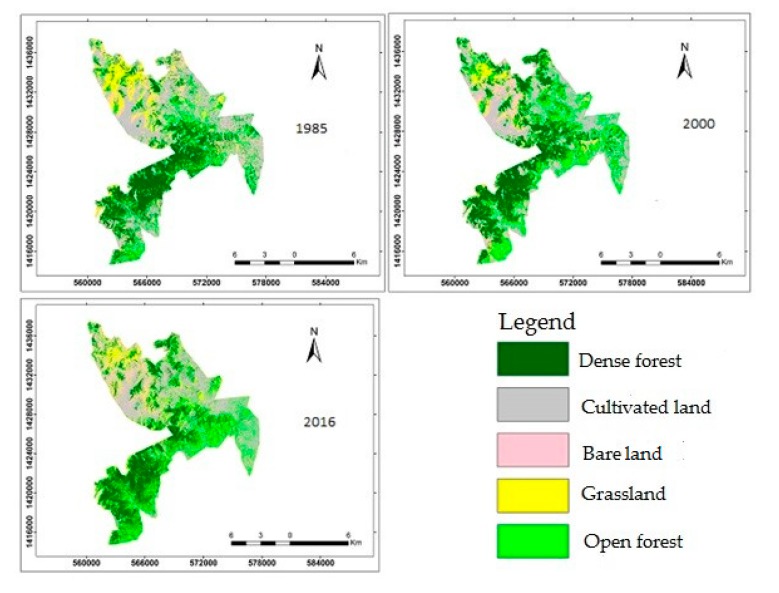
Land cover change in Wujig Mahgo Waren forest in 1985, 2000, and 2016.

The land cover data were obtained from Solomon et al. [37] (Table 1, Figure 3). The land cover data were derived from cloud-free Landsat satellite images captured in the dry season for the years 1985, 2000, and 2016 and classified using the maximum likelihood supervised classification method [37,39]. Accuracy assessment following MacLean and Congalton [40] showed that all the output maps have to meet the minimum 85% accuracy [41]. The overall accuracies for our classified thematic maps ranged from 90% to 93% with the Kappa coefficient ranging from 0.87 to 0.89 (Table 2). Change detection was conducted using a post-classification image comparison technique [42,43]. Visual comparison of features and matrix analysis were adopted to determine and detect land cover change [44]. A detailed description of the land cover data methodology is available in Solomon et al. [37].

Five land cover classes were obtained in the studied landscape (Table 1, Figure 3) [37]. In 1985, dense forest was the dominant land cover class, making up 26% of the total area, followed by bare land (24%), open forest (21%), cultivated land (19%), and grassland (10%) (Table 1, Figure 3). In 2000, dense forest constituted the largest part (28%) and open forest, bare land, cultivated land, and grassland made up 28%, 19%, 18%, and 6% of the studied landscape, respectively. Dense forest continued to be the dominant land cover class in 2016, followed by open forest, cultivated land, bare land, and grassland [37].

#### 2.2.2. Ecosystem Service Valuation

A specific set of locally relevant ecosystem services for this study was identified among the suggested ecosystem services in previous studies [17,19,30]. Ecosystem service value coefficients from TEEB valuation database [31] and locally valid value coefficients [19] were used based on the benefit transfer method to determine ecosystem service value from the five land cover categories (Table 3). The most representative biomes used as a proxy for each land cover category are: (1) tropical forest for dense forest and open forest, (2) grassland/rangeland for grassland, (3) cropland for cultivated land, and (4) desert for the bare lands. Although the represented biomes are not the same in their characteristics and functions with the land cover type in this study, they can be used as proxies for estimating ecosystem service values of the land cover types for this study area. Various studies have used a similar approach for estimating the ecosystem service values of land cover types [19,20,30,45].

In order to ensure the applicability of the transferred data from TEEB valuation database to the studied landscape conditions, values from tropical areas of land cover types similar to the geographical setting were considered. Out of the 1310 original values compiled in TEEB, 185 data points were extracted. Table 4 gives details of modified value coefficients for the ecosystem services of each land cover type. All the value coefficients were converted into 2016 USD per hectare per year to facilitate the estimation process of ecosystem value changes.

### 2.3. Estimation of Ecosystem Service Values

Ecosystem service values (ESV) from each land cover type were estimated by using the following formula following Kindu et al. [19] and Gashaw et al. [30]:

$$\mathrm{ESV}=\;\sum^{\;}\left(A_{k}\;\times {\;\mathrm{VC}}_{k}\right),$$

where A_k_ is the area (ha) and VC_k_ the value coefficient (USD ha^−1^ yr^−1^) for land cover category k. The total ESV of the entire study area was obtained by summing the estimated ESV from each land cover category. In addition to estimating land cover change effects on the total value of ecosystem services, we also estimated the impacts of such changes on individual ecosystem functions within the study area. Ecosystem service values of functions (ESV_f_) were calculated using the following equation:

$${\mathrm{ESV}}_{f}\;\left({\mathrm{USD}\;\mathrm{yr}}^{-1}\right)=\;\sum^{\;}\left(A_{k}\;\times {\;\mathrm{VC}}_{\mathrm{fk}}\right),$$

where A_k_ is the area (ha) and VC_fk_ the value coefficient of function f (USD ha^−1^ yr^−1^) for land cover category k.

The changes of ESV were obtained by calculating the difference between the estimated values in each reference year. This resulted in a summary table (Table 5) of the overall changes in ESV. The values were presented in percentages. ESV is total estimated ecosystem service value, and positive values suggest an increase whereas negative values imply a decrease in amount.

### 2.4. Estimation of Ecosystem Service Values

Considering the existing uncertainties in the value coefficients and since the biomes used as proxies for land cover types were not perfect matches in every case, sensitivity analyses were conducted to determine the percentage change in ESVs for a given percentage change in the value coefficient [46]. Accordingly, the ecosystem modified value coefficients for each land cover type were adjusted by 50% and the corresponding coefficient of sensitivity (CS) was calculated using the following equation [18,19,21]:

CS= (ESVj−ESVi)ESVi(VCj−VCi)VCik,

where ESV is the estimated ecosystem service value, VC is the value coefficient, i and j represent the initial and adjusted values, respectively, and k represents the land cover category. If the ratio of the percent change in the estimated total ESV and the percent change in the adjusted VC is greater than one, then the estimated ecosystem value is elastic with respect to that coefficient; however, if the ratio is less than one, then the estimated ecosystem value is considered to be inelastic [18]. The greater the proportional change in the ecosystem service value relative to the proportional change in the valuation coefficient, the more critical is the use of an accurate ecosystem value coefficient.

## 3. Results

### 3.1. Dynamics of Ecosystem Service Values

Table 5 presents changes in ecosystem service value for each land cover type from 1985 to 2016. The total ecosystem service values of the whole study landscape were about USD 16.6, 19.0, and 18.1 million in 1985, 2000, and 2016, respectively. In 1985, dense forest, open forest, grassland, and cultivated land accounted respectively for about 47.7%, 38.8%, 4.6%, and 8.9% of the total ecosystem service values. In 2000, dense forest contributed the largest part (i.e., 45.2%), while open forest, grasslands, and cultivated land accounted respectively for about 44.9%, 2.5%, and 7.4% of the total ecosystem service values in the study area. Dense forest, open forest, grassland, and cultivated land accounted for about 42.5%, 42.5%, 5%, and 10%, respectively, of the total ecosystem service values in 2016.

Analysis of changes in ecosystem service values revealed a gain (9.6% increase) over the whole (1985–2016) study period (Table 5). Between 1985 and 2000, total ecosystem service value increased by 15.7%. However, between 2000 and 2016, total ecosystem service values decreased by 5.2%.

**Figure 4:**
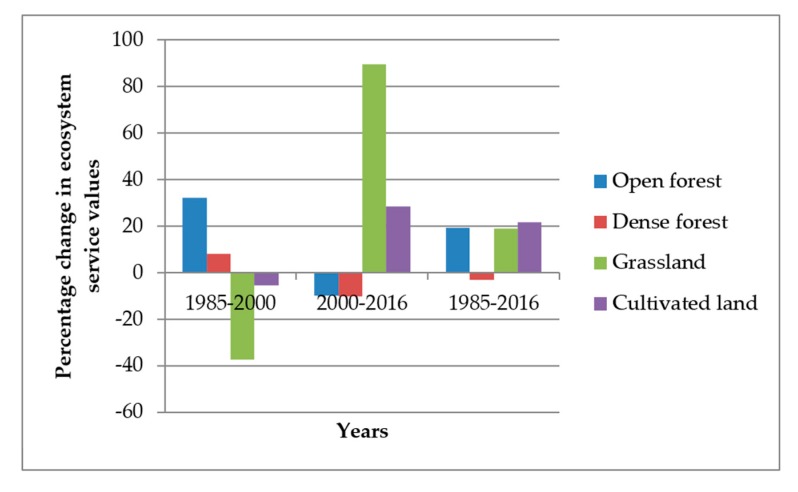
Changes in ecosystem service values between the reference years.

The impact of land cover changes on ecosystem service values differed among the land cover types, as observed in the contributions of ecosystem service values for each land cover type over the study periods (Table 5, Figure 4). The area of dense forest and open forest increased with varying proportions in the first study period (1985**–**2000). However, in the second study period (2000**–**2016) dense forest and open forest areas decreased.

### 3.2. Estimated Values of Specific Ecosystem Services and Their Changes

Table 6 indicates estimates of ecosystem service values and their changes for specific ecosystem services. In 1985, specific ecosystem service value ranged from USD 0.1 to 4.1 million, with the highest recorded for climate regulation service, and lowest for water regulation, gas regulation, and cultural services. In 2000, the ecosystem service value varied between USD 0.1 and 4.8 million, with the highest recorded in climate regulation service, and lowest in gas regulation and cultural service. In 2016, the ecosystem service value varied between USD 0.1 and 4.4 million, with the highest recorded for climate regulation service, and the lowest for water regulation and cultural service.

The highest change was observed for water regulation between 1985 and 2000 (100% increase), followed by water regulation (25% increase), disturbance regulation (20.7% increase), recreation (20.7% increase), and erosion control (19.2% increase). Between 2000 and 2016, values of water regulation (50%), recreation (8.5%), climate regulation (8.3%), genetic resources (8.3%), and erosion control (9.6%) decreased. However, pollination and food production increased by 17.6% in the same period. Between 1985 and 2016, the highest change was observed in air regulation service, with a 100% increase. Disturbance regulation and food production showed 25% and 17.6% increases in ecosystem service value in the same period, respectively.

### 3.3. Ecosystem Service Sensitivity to Value Coefficients

As indicated in Table 7, the coefficient of sensitivity was less than one in all cases. The estimated value of the ecosystem service value for the study area increased from a low of 0.05 for grassland to a high of 0.48 for a dense forest when the value coefficients for these land cover types were adjusted by 50%. Increasing or decreasing the dense forest coefficient by 50% affected the estimated 1985 ecosystem service value more (±23.8%) than the 2016 value (±21.3%). In contrast to dense forest, changing the open forest coefficient by 50% affected the estimated 1985 ecosystem service value less (±19.3%) than the 2016 value (±21.2%). Adjusting the grassland coefficient by 50% affected the estimated 1985 and 2016 ecosystem service values by ±2.3% and ±2.5%, respectively. Adjusting the cultivated land coefficient by 50% affected the estimated 1985 ecosystem service value by ±4.4% and the 2016 value by ±4.9%.

## 4. Discussion

### 4.1. Effects of Land-Use/Land-Cover Change on Ecosystem Service Values

In this study, we assessed changes in ecosystem service values in relation to land cover dynamics over the past three decades in an Afromontane forest in Northern Ethiopia. The findings reveal that total ecosystem service values increased between 1985 and 2000, and decreased between 2000 and 2016. Overall, the total estimated ecosystem service values slightly increased during the entire study period (1985–2016), resulting from the increase in open forest, grassland, and cultivated land.

Trends observed in the first period (1985–2000) could be explained by the improvement of dense forest and open forest as a result of the introduction of different interventions (e.g., intensive soil and water conservation, exclosure establishment, and community participation) [47,48,49]. The impacts of these interventions could have translated into the improvements observed between 1985 and 2000. Between 2000 and 2016, the total ecosystem service value was decreased due to the degradation of dense forest and open forest in the study area [37]. The key causes of the loss in both dense and open forest between 2000 and 2016 were fuelwood collection for household use and income generation, cultivated land expansion, population growth, free grazing, and drought [37]. Communities were encroaching on lands to get wood for fuel, construction materials, more arable land, and animal feed, which caused a slight decrease in cover between 2000 and 2016 [50,51]. This is made obvious from the results that show that the significant increase in cultivated and grassland has dictated the overall changes in the study area. The current growth of farmland and grassland at the expense of other land cover types—especially forests and woodland—in the study area may be a manifestation of the weak or inappropriate institutional arrangements in the study area, as has been observed in other parts of Ethiopia [52].

The loss of value of the ecosystem services as a result of the loss of forest cover was also revealed in various studies [19,20,21,30,45,53]. For example, Gashaw et al. [30] showed a USD 5.83 million reduction in ecosystem service value as a result of forest loss between 1985 and 2015 in the Andassa watershed in the Upper Blue Nile basin of Ethiopia. Similarly, a study by Kindu et al. [19] also reported a decline in ecosystem service value from USD 130.5 million in 1973 to USD 111.1 million in 2012 due to the loss of natural forest in the Munessa-Shashemene landscape of the Ethiopian highlands. Although these studies were conducted in different ecosystems, they provide figures which give a broad overview of the magnitude of change.

Between 1985 and 2016, the total estimated ecosystem service values showed a slight increase resulting from the increase in open forest, grassland, and cultivated land. This is because in the last 30 years the community and the government of Ethiopia have implemented several interventions to mollify the existing practice and effects of deforestation. The establishment of exclosures, the formulation of bylaws, afforestation, and the construction of soil and water conservation structures are among the main activities that have been implemented to reduce deforestation in the study area. The establishment of exclosures has been one of the strategies for rehabilitating degraded hillsides within catchments delineated for the rehabilitation and soil and water conservation programs. In line with the present study, Kassa et al. [54] and Birhane et al. [55] have stated that since the 1980s, area exclosure has been introduced to manage degraded lands and increase productivity in Northern Ethiopia. In addition, community members work to enrich the forest land by planting seedlings which provide ecological and economic benefits. This result is supported by Birhane et al. [55], who discovered that community members in the Tigray region of Northern Ethiopia had participated in enrichment planting programs to improve their forest cover. Inside the forest, different soil and water conservation activities have been carried out to reduce soil erosion and to increase the moisture content of the land so that seed banks easily regenerate, eventually increasing the forest cover. This was common practice in different areas of the country. For instance, Birhane et al. [55], who studied exclosures as a forest restoration tool, indicated that the construction of soil and water conservation structures is one of the measures practiced by the community to rehabilitate degraded lands in Northern Ethiopia. Seyoum et al. [56] also reported that massive soil and water conservation programs were carried out to restore degraded lands in Northern Ethiopia.

In a similar study by Temesgen et al. [57], ecosystem service value increased from USD 129 millions to USD 147 millions between 1986 and 2015 in the agroforestry-dominated landscape in Ethiopia.. Such increases in ecosystem service values are also evident in different parts of the world. For example, Arowolo et al. (2018) reported an increase in the total ecosystem services value in Nigeria from 665.93 billion (2007 USD) in 2000 to 667.44 billion (2007 USD) in 2010 due to expansion of cultivated land. Wang et al. [58] found that ecosystem service value increased from USD 1.82 billion to 2.24 billion between 2000 and 2010 in Ningxia, China, which was ascribed to an increase in forest and grasslands. Similarly, Camacho-Valdez et al. [23] reported that total ecosystem service value increased by approximately 9% between 2000 and 2010 in the southern coast of Sinaloa State, Northwest Mexico. This contrasts with findings of Kindu et al. [19] and Gashaw et al. [30]. Contrary to the present study, Gashaw et al. [30] showed a USD 5.83 million reduction in ecosystem service value as a result of forest loss between 1985 and 2015 in the Andassa watershed in the Upper Blue Nile basin of Ethiopia. Similarly, a study by Kindu et al. [19] also reported a decline in ecosystem service from USD 130.5 million in 1973 to USD 111.1 million in 2012 due to the loss of natural forest in the Munessa-Shashemene landscape of the Ethiopian highlands. Tolessa et al. [53] reported a reduction in the overall ecosystem services value by 68% between 1973 and 2014 in the central highlands of Ethiopia, and this was mainly due to deforestation. Such decreases in ecosystem service values are also evident in different parts of the world. For example, in Mozambique, Niquisse and Cabral [59] estimated a decline in ecosystem service value by 11.4% between 2005 and 2009. For example, in Chongming Island, China, Zhao et al. [60] estimated a decline in ecosystem service value by 62% between 1990 and 2000. Li et al. [46] also showed around 5% decrease in ecosystem service value from 1975 to 2005 in Zoige Plateau, China.

The main contributor to ecosystem service values across the study periods was the forest ecosystems (open and dense forests), though the contribution of each land cover type to the ecosystem service value varied. Kindu et al. [19] also found higher ecosystem services in natural forest of the Munessa-Shashemene landscape of the Ethiopian highlands. As compared to the forest ecosystem, the contribution of grassland and cultivated land were negligible in the present study.

Between 2000 and 2016, values of water regulation, recreation, climate regulation, genetic resources, and erosion control services decreased as a result of the decline in dense forest and open forest in the study area. This was in agreement with the study conducted by Wang et al. [61], who observed a reduction in the value of air purification, climate regulation, and other ecosystem services due to a decline in the area of grasslands, woodlands, and aquatic regions between 2003 and 2013 in Manas river basin, China. In a similar study by Kindu et al. [19], water regulation, recreation, climate regulation, genetic resources, and erosion control services decreased between 1973 and 2012 because of the loss of natural forests, woodlands, shrubland, and grasslands in the Munessa-Shashemene landscape of the Ethiopian highlands. Gashaw et al. [30] also showed a reduction in several individual ecosystem service values between 1985 and 2015 due to forest and shrubland degradation in the Andassa watershed of Ethiopia.

On the other hand, food production service value increased between 2000 and 2016, which was mainly the result of an increase in the cultivated land within the study area. Similarly, in a study of the Munessa-Shashemene landscape of the Ethiopian highlands, Kindu et al. [19] reported that food production services increased between 1973 and 2012 while other ecosystem services such as disturbance regulation, cultural, and recreational ecosystem services decreased due to the expansion of cultivated land. Tolessa et al. [53] also found an improvement in food production services, while other ecosystem services such as raw material production, recreation, and cultural services declined in the central highlands of Ethiopia. Gashaw et al. [30] also observed an increase in food production, biological control and pollination in the Andassa watershed of Ethiopia.

### 4.2. Ecosystem Service Sensitivity Analysis

The method used to estimate ecosystem service value was derived by multiplying the area of a given land use category by the corresponding value coefficient. The values produced using this method have low resolution, high deviation, and high uncertainly due to the unpredictable, dynamic, and nonlinear properties of biological systems [30,62,63]. Land use can be used as a proxy for ecosystem services, but the biomes used as proxies are not always perfect matches [18]. However, the results of the sensitivity analysis indicated that the estimated ecosystem service values for the study landscape were relatively inelastic with respect to the value coefficients, and that the estimates were relatively robust despite uncertainties in the value coefficients. The evaluation is thus valid for calculating ecosystem service values over extended periods as a means of assessing their changes in response to land-use/land-cover dynamics. This has also been highlighted in previous studies in various contexts [19,53,60].

## 5. Conclusions

This study provided empirical evidence on the impacts of land-use/land-cover dynamics from 1985 to 2016 on ecosystem service values in dry Afromontane forest ecosystems in Northern Ethiopia. It showed that although a slight gain was estimated for the entire study period, ecosystem service values decreased from 2000 to 2016. The findings revealed that values of water regulation, recreation, climate regulation, genetic resources, and erosion control services decreased as a result of the decline in dense forest and open forest in the study area. However, food production service value increased between 2000 and 2016, which was mainly the result of an increase in the cultivated land within the study area.

This study highlighted how forest cover dynamics can be reflected or translated in changes in ecosystem service values in dry Afromontane forest ecosystems in Northern Ethiopia. The findings can serve as crucial inputs for policy and strategies formulations for the sustainable use and management of forest resources in Northern Ethiopia. The findings can also be very useful to raise awareness on the importance and need for the conservation of forest resources. It can also guide the allocation of limited resources among competing demands to safeguard the ecosystems that offer the best-valued services. Furthermore, since the ecosystem service values of the landscape have been decreasing in recent years (i.e., from 2000 to 2016), developing scenarios, though modelling, of future direction and changes in ecosystem service value could help guide decisions on sustainable management of the resources to maintain and ensure the current and future provision of ecosystem services.

## Figures and Tables

**Table 1:** Area and proportion of land cover change in Wujig Mahgo Waren forest in 1985, 2000, and 2016.

Land Cover Types	Land Cover Distribution						Land Cover Change Dynamics (%)		
	1985		2000		2016		1985–2000	2000–2016	1985–2016
LC Types	Area (ha)	%	Area (ha)	%	Area (ha)	%			
Dense forest	4469	26	4836	28	4335	25	8	−10	−3
Open forest	3629	21	4802	28	4337	25	32	−10	16
Grassland	1713	10	1074	6	2035	12	−37	90	16
Cultivated land	3211	19	3035	18	3902	23	−6	29	18
Bare land	3999	24	3272	19	2417	14	−18	−26	−65

Source: Solomon et al. [37].

**Table 2:** Accuracy assessment of classified images.

Land Cover Types	Accuracy (%)					
	1985		2000		2016	
	Producer’s	User’s	Producer’s	User’s	Producer’s	User’s
Open forest	77	95	85	90	81	91
Dense forest	97	91	98	89	94	93
Cultivated land	95	90	95	91	97	84
Bare land	85	100	79	95	69	90
Grassland	89	93	82	90	96	93
Overall Accuracy	93		90		90	
Kappa Coefficient	0.89		0.87		0.87	

Source: Solomon et al. [37].

**Table 3:** Land cover types and biome equivalents with the corresponding value coefficients.

Land Cover Categories	Equivalent Biome	Ecosystem Service Coefficient (USD ha^−1^ yr^−1^)
**Dense forest**	Tropical forest	1775
**Open forest**	Tropical forest	1775
**Grassland**	Grass/rangelands	447
**Cultivated land**	Cropland	463
**Bare lands**	Desert	0

Source: Computed from The Economics of Ecosystem and Biodiversity valuation database (TEEB) [31] and Kindu et al. [19].

**Table 4:** Details of annual value coefficients for ecosystem service functions of each land cover type.

Ecosystem Services	Ecosystem Service Values (USD ha^-1^ yr^-1^) of Land Cover Types			
	Dense forest	Open forest	Grassland	Cultivated land
**Provisioning services**				
Water supply	41	41	72	
Food production	57	57	159	310
Raw material	110	110	21	43
Genetic resources	103	103	18	51
**Regulating services**				
Water regulation	15	15	7	
Waste treatment	201	201	50	
Erosion control	320	320	23	5
Climate regulation	482	482	51	20
Biological control	5	5	30	30
Air regulation	15	15	10	
Disturbance regulation	53	53		
**Cultural services**				
Cultural	11	11		
Recreation	362	362	6	4
**Total**	1775	1775	447	463

Source: Computed from The Economics of Ecosystem and Biodiversity valuation database (TEEB) [31] and Kindu et al. [19].

**Table 5:** Estimated ecosystem service values and dynamics for each land cover type of the different reference years and periods in million USD per year of the study area.

Land Cover Types	Ecosystem Service Values						Ecosystem Service Value Changes (%)		
	1985		2000		2016		1985–2000	2000–2016	1985–2016
	USD	%	USD	%	USD	%			
Dense forest	7.9	47.7	8.6	45.2	7.7	42.5	8.2	−10.4	−3.0
Open forest	6.4	38.8	8.5	44.9	7.7	42.5	32.3	−9.8	19.3
Grassland	0.77	4.6	0.48	2.5	0.9	5.0	−37.3	89.5	18.8
Cultivated land	1.49	8.9	1.4	7.4	1.8	10.0	−5.5	28.6	21.5
Bare land	0	0	0	0	0	0	0	0	0
Total	16.6	100	19.0	100	18.1	100	15.7	−5.2	9.6

**Table 6:** Annual estimated value of ecosystem functions (ESV_f_ in million USD year^−1^) under each service category for different reference years and their changes (1985 to 2016).

Ecosystem Services	Ecosystem Service Value (ESV) Million USD Year^−1^			Ecosystem Service Value Change (%)		
	ESV1985	ESV2000	ESV2016	1985–2000	2000–2016	1985–2016
**Provisioning services**						
Water supply	0.5	0.5	0.5	0	0	0.0
Food production	1.7	1.7	2.0	0	17.6	17.6
Raw material	1.1	1.2	1.2	9	0	9.0
Genetic resources	1.0	1.2	1.1	20	−8.3	10.0
**Regulating services**						
Water regulation	0.1	0.2	0.1	100	−50	0.0
Water treatment	1.7	2.0	1.8	17.6	−10	5.9
Erosion control	2.6	3.1	2.8	19.2	−9.6	7.7
Climate regulation	4.1	4.8	4.4	17.2	−8.3	7.3
Biological control	0.2	0.2	0.2	0	0	0.0
Air regulation	0.1	0.1	0.2	0	1	100
Disturbance regulation	0.4	0.5	0.5	25	0	25
**Cultural services**						
Cultural	0.1	0.1	0.1	0	0	0.0
Recreation	2.9	3.5	3.2	20.7	−8.5	10.3
Total	16.5	19.1	18.1	15.7	−5.2	9.6

**Table 7:** Percentage change in the estimated total ecosystem service value (ESV) and coefficient of sensitivity (CS) resulting from a 50% adjustment in the ecosystem valuation coefficients (VCs).

Change in Valuation Coefficient (VC)	The Effect of Changing VC from the Original Value			
	1985		2016	
	%	CS	%	CS
Dense forest VC ± 50%	±23.8	0.48	±21.3	0.43
Open forest VC ± 50%	±19.3	0.40	±21.2	0.42
Grassland VC ± 50%	±2.3	0.05	±2.5	0.05
Cultivated land VC ± 50%	±4.4	0.09	±4.9	0.1
